# Dynamic Social Community Detection and Its Applications

**DOI:** 10.1371/journal.pone.0091431

**Published:** 2014-04-10

**Authors:** Nam P. Nguyen, Thang N. Dinh, Yilin Shen, My T. Thai

**Affiliations:** Department of Computer and Information Science and Engineering, University of Florida, Gainesville, Florida, United States of America; Universidad de Zarazoga, Spain

## Abstract

Community structure is one of the most commonly observed features of Online Social Networks (OSNs) in reality. The knowledge of this feature is of great advantage: it not only provides helpful insights into developing more efficient social-aware solutions but also promises a wide range of applications enabled by social and mobile networking, such as routing strategies in Mobile Ad Hoc Networks (MANETs) and worm containment in OSNs. Unfortunately, understanding this structure is very challenging, especially in dynamic social networks where social interactions are evolving rapidly. Our work focuses on the following questions: How can we efficiently identify communities in dynamic social networks? How can we adaptively update the network community structure based on its history instead of recomputing from scratch? To this end, we present Quick Community Adaptation (QCA), an adaptive modularity-based framework for not only discovering but also tracing the evolution of network communities in dynamic OSNs. QCA is very fast and efficient in the sense that it adaptively updates and discovers the new community structure based on its history together with the network changes only. This flexible approach makes QCA an ideal framework applicable for analyzing large-scale dynamic social networks due to its lightweight computing-resource requirement. To illustrate the effectiveness of our framework, we extensively test QCA on both synthesized and real-world social networks including Enron, arXiv e-print citation, and Facebook networks. Finally, we demonstrate the applicability of QCA in real applications: (1) A social-aware message forwarding strategy in MANETs, and (2) worm propagation containment in OSNs. Competitive results in comparison with other methods reveal that social-based techniques employing QCA as a community detection core outperform current available methods.

## Introduction

Many social networks in practice commonly exhibit the property of containing community structure [1], [2], i.e., they naturally divide into groups of nodes with denser connections inside each group and fewer connections crossing between groups. In general, nodes and connections in a social network typically represent network users and their social interactions (e.g., friendships in Facebook, following in Twitter or professional connections in LinkedIn), respectively. Members in each social community typically have some certain interests in common such as photography, movies, music or travel, and hence, they tend to interact more frequently with each other than with users who are outside of their community. Community detection in a social network, as a result, is the gathering of its users into groups in such a way that nodes in each group are densely connected inside and sparser outside.

Community detection and graph clustering problem are closely related to each other due to their nature. Nevertheless, it is noteworthy to differentiate between them. While these two problems share the same objective of partitioning network nodes into groups, the number of clusters in graph clustering is often predefined (or given as a part of the input) whereas the number of communities is typically unknown in community detection. In the visualization perspective, communities display the whole network organization as a compact and more understandable level where each community can represent a functional group or an entity in the system. At this level, community structure provides us meaningful insights into network's organizational principles, and consequently, sheds light on preventing potential vulnerability and security threats such as network corruption and computer virus and worm propagation [3]. Studies on community detection on static networks can be found in an excellent survey [4], as well as in the work of [5]–[7] and references therein.

Real-world social networks, however, are not always static. In fact, most popular social sites in reality (such as Facebook, Twitter and LinkedIn) evolve heavily and witness a rapid expansion in terms of size and space over time. As a result, they lend themselves naturally to the field of dynamic networks. A dynamic network is a special type of evolving complex graphs in which changes are frequently introduced over time. In the senses of OSNs, these changes are commonly introduced by users joining in or withdrawing from one or more communities, by friends and friends connecting together, or by new users making friend with one another. Although any of these social events seems to have a little effect to a local structure of the network on one hand; the network's dynamics over a long duration on the other hand, may lead to a significant transformation of the entire community structure, and consequently raise a natural need of reidentification. However, the rapid and unpredictable changing topological structures of dynamic social networks makes it an extremely complicated yet challenging problem.

**Figure 1:**
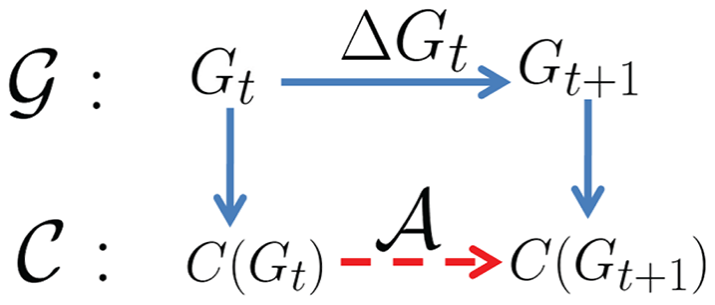
The overview of our adaptive community structure detection algorithm.

Although one can certainly execute one of the available static community detection methods [5], [8]–[10] all over again to find the new structure whenever the network evolves, he may encounter some disadvantages that cannot be neglected (1) the expensive execution time of the specific method on large networks, (2) the trap of local optima, and (3) the almost same reaction to a small change to some local parts of the network. A better, much efficient and less time consuming approach to accomplish this expensive task is to adaptively update the network communities from the previously discovered structures, which obscure the hassle of repeatedly recomputation from scratch. This adaptive approach is the main focus of our study in this paper. In Figure 1, we briefly generalize the idea of community structure adaptation in an evolving network: the network evolves from time *t* to *t*+1 under the change Δ*G_t_*. The adaptive algorithm 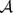 quickly finds the updated community structure 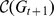 based on the previous structure 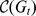 together with the changes Δ*G_t_*.

In an application perspective, the detection of communities in a dynamic social network is of considerable advantages. To give a sense of its effects, let us consider the routing problem in communication network where nodes and links represent people and mobile communications, respectively. Due to nodes' mobility and unstable links properties of the network, designing an efficient routing scheme is extremely challenging. However, since people have a natural tendency to form groups of communication, there exist groups of densely connected nodes in the underlying MANET as a reflection, and hence, forms community structure in that MANET. An effective routing algorithm, as soon as it discovers the network communities, can directly route or forward messages to nodes in the same or a related community as the destination. By doing in this way, we can avoid unnecessary messages forwarding through nodes in different communities, and therefore can lower down the number of duplicate messages and overhead information, which are essential factors in MANETs.

The contributions of this paper are threefold. First, we propose QCA, a fast adaptive framework for efficiently identifying the disjoint community structure of dynamic social networks. Our approach takes into account the structural history and works on network changes only, thus significantly reduces computational cost and time requirement. We also carry out theoretical results regarding communities' behaviors over time, which are the fundamentals of our method. Second, we extensively evaluate the proposed framework on both synthesized and real dynamic social traces. Experimental results show that QCA achieves not only competitive modularity scores but also high quality community structures in a timely manner. Finally, we apply QCA method to two practical applications: forwarding strategies in MANETs and worm containment in OSNs. Simulation results show that strategies utilizing QCA outperform current available methods and confirm its applicability in social network problems.

## Preliminaries

In this section we first present the graph notations that will be used throughout the paper. We then formulate the dynamic social network, the objective function and finally the problem definition based on the defined notations.

### Notations

Let *G* = (*V*, *E*) be an undirected and unweighted graph representing a social network with *N* nodes and *M* edges. Let 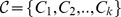 denote a disjoint partitioning of *V*, where 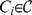 is a community of *G*. For each vertex 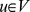, its degree, the community containing *u* and the set of its adjacent communities are respectively denoted by 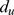, 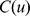 and 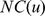. For any 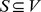, let 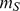, 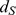, and 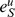 be the number of links inside *S*, the total degree of vertices in *S*, and the number of connections from *u* to *S*, respectively. The pairs of terms *node* and *vertex*, as well as *edge* and *link* and are used interchangeably.

### Dynamic networks

Let 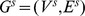 be a time dependent network snapshot recorded at time *s*. Denoted by 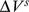 and 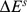 the sets of vertices and links to be introduced (or removed) at time *s*, and let 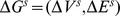 denote the change in terms of the whole network. The next network snapshot 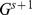 is expressed as 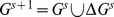. A *dynamic social network* 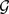 is a sequence of network snapshots evolving over time: 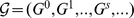.

### Objective function

To quantify the quality of a detected network community structure, we use the widely accepted measure called *modularity* 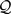 [11], defined as
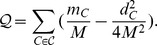
Generally, 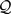 is the fraction of all links within communities less the expected value of the same quantity in a graph whose nodes have the same degrees but links are distributed randomly, and the higher modularity 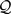, the better network community structure is. Hence, our objective is to find a community assignment for network vertices so that 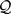 is maximized.

### Problem Definition

Given a dynamic social network 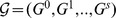 where 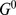 is the original network and 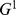, 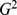,…, 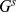 are the network snapshots obtained through 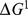, 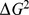,…, 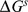, we need to devise adaptive algorithms to efficiently identify the network community structure at any time point as well as to trace the evolution of the network communities.

## Methods

Let us first discuss how changes introduced to the evolving network topology affect the structure of its communities. We use the term *intra-community links* to denote edges whose two endpoints belong to the same community, and the term *inter-community links* to denote those with endpoints connecting different communities. For each community *C*, the connections linking *C* with other communities are much fewer than those within *C* itself, i.e., nodes in *C* are densely connected inside and sparsely connected outside. Intuitively, adding intra-community links inside or removing inter-community links between communities of *G* will strengthen those communities and make the structure of *G* more clear. Vice versa, removing intra-community links and inserting inter-community links will loosen the structure of *G*. However, when two communities have less distraction caused by each other, adding intra or removing inter-community links makes them more attractive to each other and thus, leaves a possibility that they will be combined to form a new community. The community updating process, as a result, is challenging since an insignificant change in the network topology can possibly lead to an unexpected transformation of its community structure. We will discuss in detail the possible behaviors of dynamic network communities in the following subsections.

In order to reflect changes introduced to a social network, its underlying graph is frequently updated by either inserting or removing a node or a set of nodes, or by either introducing or deleting an edge or a set of edges. In fact, the introduction or removal of a set of nodes (or edges) can be decomposed as a sequence of node (or edge) insertions (or removals), in which a single node (or a single edge) is introduced (or removed) at a time. This observation helps us to treat network changes as a collection of *simple events* where a simple event can be one of *newNode*, *removeNode*, *newEdge*, *removeEdge* whose details are as follow:

- *newNode* 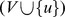: A new node *u* together with its associated edges are introduced. *u* could come with no or more than one new edge(s).
- *removeNode* 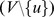: A node *u* and its adjacent edges are removed from the network.
- *newEdge* 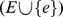: A new edge *e* connecting two existing nodes is introduced.
- *removeEdge* 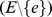: An existing edge *e* in the network is removed.

### Algorithms

Our approach first requires an initial community structure 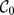, which we call the *basic structure*, in order to process further. Since the input model is restricted as an undirected and unweighted network, this initial community structure can be obtained by performing any of the available static community detection methods [5], [8], [9]. To obtain a good basic structure, we choose the method proposed by Blondel et al. [8] which produces a good network community structure in a timely manner [4].

#### New node

Let us consider the first case when a new node *u* and its associated connections are introduced. Note that *u* may come with no adjacent edges or with many of them connecting one or more communities. If *u* has no adjacent edge, we create a new community for it and leave the current structure intact. The interesting case happens, and it usually does, when *u* comes with edges connecting one or more existing communities. In this latter situation, we need to determine which community *u* should join in, or which nodes in other communities that should together with *u* form a new community in order to maximize the gained modularity. In addition, the introduction of *u* might cause some part of an existing community to leave its current host and move to another community. To handle this case, we first determine whether any neighbor node of *u* should change its community membership or not. There are several local methods introduced for this task, for instance the algorithms of [5], [9]. Our method is inspired by a physical approach proposed in [12], in which each node is influenced by two forces: 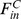 (to keep *u* stays inside community *C*) and 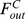 (the force a community *C* makes in order to bring *u* to *C*) defined as follow:
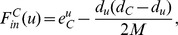
and
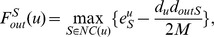
where 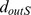 is of opposite meaning of 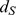.

Taking into account the above two forces, we first determine whether a node *u* should form a new community with other nodes in its neighbor communities. This is done by iteratively selecting nodes that are more attracted by *C*(*u*) rather than its current community (the outer “while” loop in Table 1 Algorithm 1). Otherwise, node *u* can actively determines its best community membership by computing those forces and either lets itself join the community *S* having the highest 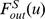 (if 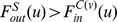) or stays in the current community *C*(*v*) otherwise. By Proposition 1, we bridge the connection between those forces and the objective function, i.e., joining the new node in the community with the highest outer force will maximize the local gained modularity. The process is presented in Table 1 Algorithm 1.

**Table 1:** Algorithm 1. New_Node.

**Input:** New node *u* with associated links; Current structure 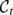 .
**Output:** An updated structure 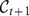
1: *C*(*u*) ← A new community of only *u*;
2: *Done* ← *False*;
3: **while** (!*Done*) **do**
4: **for** (*v* ∈ *N*(*u*) and *v* is not visited) **do**
5: Find 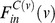 and 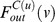 ;
6: **end for**
7: Sort *v* ∈ *NC*(*u*) by its 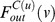 given 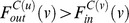 .
8: Let **v** be the stack containing these sorted nodes;
9: **if** ( 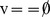 ) **then**
10: *Done* ← *True*;
11: **end if**
12: *C*(*u*) ← *C*(*u*) ∪ {*pop*(**v**)};
13: Marked *v* as visited;
14: **end while**
15: **for** *C* ∈ *NC*(*u*) **do**
16: Find 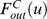 ;
17: **end for**
18: **if** max*_C_* 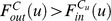 **then**
19: Let 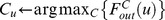 ;
20: Update 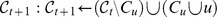 ;
21: **end if**

**Proposition 1.** *Let C be the community having the maximum* 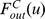 *when a new node u with degree p is added to G, then joining u in C gives the maximal gained modularity (Note: All proofs are included in the Appendix).*

*Proof.* Let *D* be a community of *G* and *D*≠*C*, we show that joining *u* in *D* contributes less modularity than joining *u* in *C*. The overall modularity 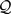 when *u* joins in *C* is
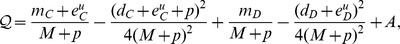
where *A* is the summation of other modularity contributions. Similarly, joining *u* to *D* gives
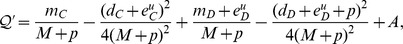
and
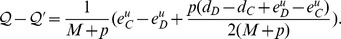
Now, since *C* is the community that gives the maximum 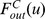, we obtain
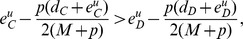
which implies
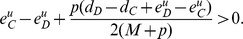
Hence, 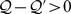 and thus the conclusion follows.□

#### New edge

When a new edge 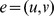 connecting two existing vertices 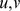 is introduced, we divide it further into two subcases: *e* is an intra-community link (totally inside a community *C*) or an inter-community link (connects two communities *C*(*u*) and *C*(*v*)). If *e* is inside a community *C*, its presence will strengthen the internal modularity structure of *C* according to Proposition 2. Furthermore, by Proposition 3, we know that adding *e* should not split the current community *C* into smaller modules. Therefore, we leave the current network structure intact in this case.

**Figure 2:**
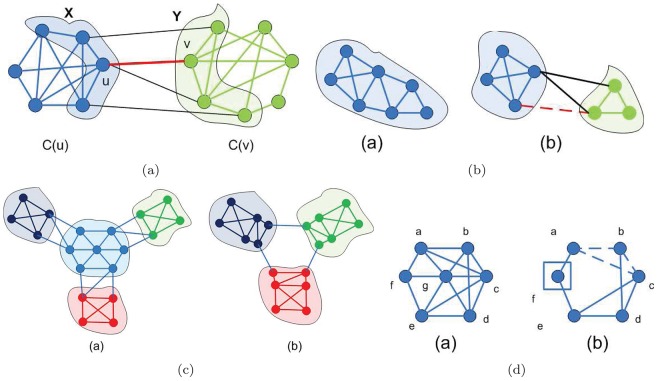
Possible behaviors of the dynamic community structure.

The interesting situation occurs when *e* is a link connecting communities *C*(*u*) and *C*(*v*) since its presence could possibly make *u* (or *v*) leave its current module and join in the new community. Additionally, if *u* (or *v*) decides to change its membership, it can advertise its new community to all its neighbors and some of them might eventually want to change their memberships as a consequence. By Proposition 4, we show that should *u* (or *v*) ever change its community assignment, *C*(*v*) (or *C*(*u*)) is the best new community for it. But how can we quickly decide whether *u* (or *v*) should change its membership in order to form a better community structure with higher modularity? To this end, we provide a criterion to test for membership changing of *u* and *v* in Proposition 5. Here, if both 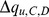 and 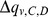 fail to satisfy the criteria, we can safely preserve the current network community structure (Corollary 1). Otherwise, we move *u* (or *v*) to its new community and consequently let its neighbors determine their best modules to join in, using local search and swapping to maximize gained modularity. Figure 2a describes the procedure for this latter case. The detailed algorithm is described in Table 2 Algorithm 2.

**Table 2:** Algorithm 2. New_Edge.

**Input:** Edge {*u*, *v*} to be added; Current structure 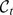 .
**Output:** An updated structure 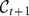 .
1: **if** (*u* and 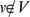 ) **then**
2: 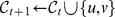 ;
3: **else if** (*C*(*u*)≠*C*(*v*)) **then**
4: **if** (Δ*q_u_*_,*C*(*u*),*C*(*v*)_<0 and Δ*q_v_*_,*C*(*u*),*C*(*v*)_<0) **then**
5: **return** 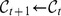 ;
6: **else**
7: *w* ← arg max{Δ*q_u_*_,*C*(*u*),*C*(*v*)_, Δ*q_v_*_,*C*(*u*),*C*(*v*)_};
8: Move *w* to the new community;
9: **for** (*t* ∈ *N*(*w*)) **do**
10: Let *t* determine its best community;
11: **end for**
12: Update *C_t_*_+1_;
13: **end if**
14: **end if**

**Proposition 2.** *For any* 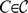 *, if* 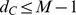 *then adding an edge within C will increase its modularity contribution.*

*Proof.* The portion 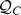 that community *C* contributes to the overall modularity 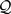 is:
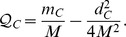
When a new edge coming in, the new modularity 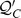 is
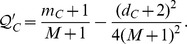
Taking the difference between the two expressions 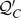 and 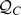 gives
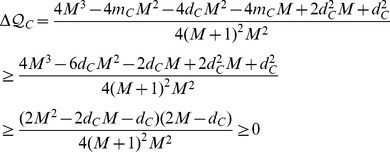
The last inequality holds since 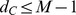 implies 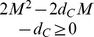.□

**Proposition 3.** *If C is a community in the current snapshot of G, then adding any intra-community link to C should not split it into smaller modules.*

*Proof.* Assume the contradiction, i.e, *C* should be divided into smaller modules when an edge is added into it. Let 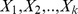 be disjoint subsets of *C* representing these modules. Let *d_i_* and *e_ij_* be the total degree of vertices inside *X_i_* and the number of links going from *X_i_* to *X_j_*, respectedly. Assume that, W.L.O.G., when an edge is added inside *C*, it is added to *X*_1_.

Recall that
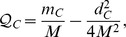
and
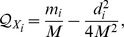
(where *m_i_* is short for 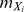). Prior to adding an edge to *C*, we have
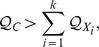
or equivalently,
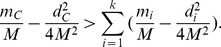
Since 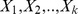 are disjoint subsets of *C*, it follows that 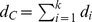 and
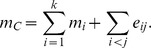
The above inequality equals to
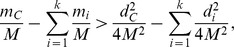
or
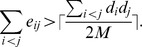
Now, assume that the new edge is added to *X*_1_ and *C* is split into 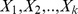 which implies that dividing *C* into *k* smaller communities will increase the overall modularity, i.e, 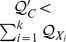. This implies that
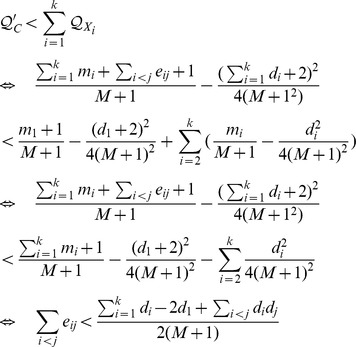
Since 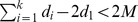, we have
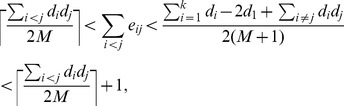
and thus the conclusion follows.□

**Proposition 4.** *When a new edge* 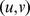 *connecting communities* 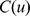 *and* 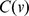 *is introduced,* 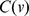 *(or* 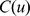 *) is the best candidate for u (or v) if it should ever change its membership.*

*Proof.* Let 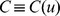 and 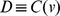. Recall the outer force that a community *S* applies to vertex *u* is
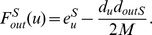
We will show that the presence of edge 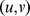 will strengthen 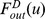 while weakening the other outer forces 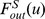, i.e, we show that 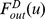 increases while 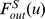 decreases for all 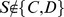.
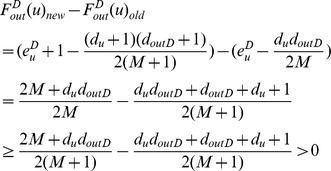
and thus 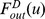 is strengthened when 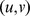 is introduced. Furthermore, for any community 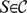 and 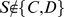,
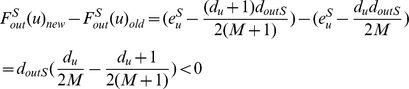
which implies 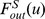 is weakened when 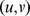 is connected. Hence, the conclusion follows.□

**Proposition 5.** *Assume that a new edge* 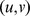 *is added to the network. Let* 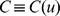 *and* 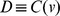 *. If* 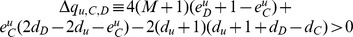 *then joining u to D will increase the overall modularity.*

*Proof.* Node *u* should leave its current community *C* and join in *D* if
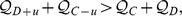
or equivalently,
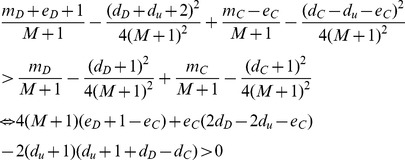
□

**Corollary 1.** *If the condition in Proposition 5 is not satisfied, then neither u nor its neighbors should be moved to D.*

#### Node removal

When an existing node *u* in a community *C* is removed, all of its adjacent edges are disregarded as a result. This case is challenging in the sense that the resulting community is very complicated: it can be either unchanged or broken into smaller pieces and could probably be merged with other communities. Let us consider two extreme cases when a single degree node and a node with highest degree in a community is removed. If a single degree node is removed, it leaves the resulted community unchanged (Proposition 7). However, when a highest degree vertex is removed, the current community might be disconnected and broken in to smaller pieces which then are merged to other communities as depicted in Figure 2c. Therefore, identifying the leftover structure of *C* is a crucial part once a vertex in *C* is removed.

To quickly and efficiently handle this task, we utilize the clique percolation method presented in [2]. In particular, when a vertex *u* is removed from *C*, we place a 3-clique to one of its neighbors and let the clique percolate until no vertices in *C* are discovered (Figure 2d). We then let the remaining communities of *C* choose their best communities to merge in. The detailed algorithm is presented in Table 3 Algorithm 3.

**Table 3:** Algorithm 3. Node_Removal.

**Input:** Node *u* ∈ *C* to be removed; Current structure 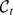 .
**Output:** An updated structure 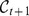 .
1: *i* ← 1;
2: **while** ( 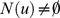 ) **do**
3: *S_i_* ←{Nodes found by a 3-clique percolation on *v* ∈ *N*(*u*)};
4: **if** ( 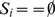 ) **then**
5: *S_i_* ← {*v*};
6: **end if**
7: *N*(*u*) ← *N*(*u*)\*S_i_*;
8: *i* ← *i*+1;
9: **end while**
10: Let each singleton in *N*(*u*) consider its best communities;
11: Let each *S_i_* consider its best communities as in [8]
12: Update 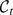 ;

#### Edge removal

In the last case when an edge 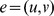 is removed, we divide further into four subcases: (1) *e* is a single edge connecting only *u* and *v*, (2) either *u* or *v* has degree one, (3) *e* is an inter-community link connecting *C*(*u*) and *C*(*v*), and (4) *e* is an intra-community link. If *e* is an single edge, its removal will result in the same community structure plus two singletons of *u* and *v* themselves. The same reaction applies to the second subcase when either *u* or *v* has single degree due to Proposition 7, thus results in the prior network structure plus *u* (or *v*). When *e* is an inter-community link, the removal of *e* will strengthen the current network communities (Proposition 6) and hence, we just make no change to the overall network structure.

The last but most complicated case happens when an intra-community link is deleted. As depicted in Figure 2b, removing this kind of edge often leaves the community unchanged if the community itself is densely connected; however, the target module will be divided if it contains substructures which are less attractive or loosely connected to each other. Therefore, the problem of identifying the structure of the remaining modules is important. Proposition 9 provides us a convenient tool to test for community bi-division when an intra-community link is removed from the host community *C*. However, it requires an intensive look for all subsets of *C*, which may be time consuming when *C* is big. Note that prior to the removal of 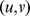, the community *C* hosting this link should contain dense connections within itself and thus, the removal of 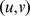 should leave some sort of ‘quasi-clique’ structure [2] inside *C*. Therefore, we find all maximal quasi-cliques within the current community and have them (as well as leftover singletons) determine their best communities to join in. The detailed procedure is described in Table 4 Algorithm 4.

**Table 4:** Algorithm 4. Edge_Removal.

**Input:** Edge (*u*, *v*) to be removed; Current structure 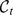 .
**Output:** An updated clustering 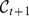 .
1: **if** ((*u*, *v*) is a single edge) **then**
2: 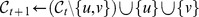 ;
3: **else if** (Either *u* (or *v*) is of degree one) **then**
4: 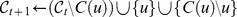 ;
5: **else if** (*C*(*u*)≠*C*(*v*)) **then**
6: 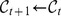 ;
7: **else**
8: % Now (*u*, *v*) is inside a community *C* %
9: *L* = {Maximal quasi-cliques in *C*};
10: Let the singletons in *C*\*L* consider their best communities;
11: **end if**
12: Update 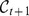 ;

**Proposition 6.** *If* 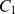 *and* 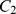 *are two communities of G, then the removal of an inter-community link connecting them will strengthen modularity contributions of both* 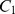 *and* 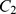 *.*

*Proof.* Let 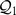 and 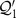 be the modularities of 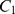 before and after the removal of that link. We show that 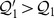 (and similarly, 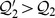) and thus, 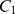 and 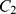 contribute higher modularities to the network. Now,
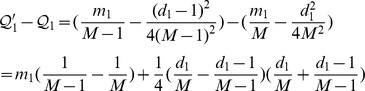
Since all terms are all positive, 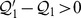. The same technique applies to show that 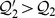.□

**Proposition 7.** *The removal of* 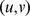 *inside a community C where only u or v is of degree one will not separate C.*

*Proof.* The proof of this proposition is similar to that of proposition 3.□

**Proposition 8.** *(Separation of a community) Let* 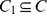 *and* 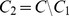 *be two disjoint subsets of C.* 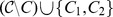 *is a community structure with higher modularity when an edge crossing* 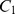 *and* 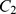 *is removed, i.e., C should be separated into* 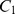 *and* 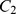 *, if and only if* 
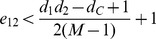
 *.*

*Proof.* Let 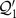, 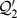 and 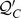 denote the modularity contribution of 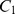, 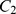 and *C* after an edge crossing 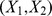 has been removed. Now,
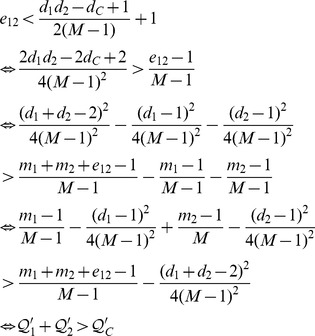
Thus, the conclusion follows.□

**Proposition 9.** *(Community bi-division) For any community C, let α and β be the lowest and the second highest degree of vertices in C, respectively. Assume that an edge e is removed from C. If there do not exist subsets* 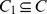 *and* 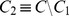 *such that e is crossing* 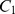 *and* 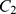 *and* 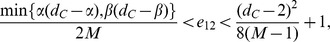 *then any bi-division of C will not benefit the overall* 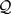 *.*

*Proof.* From Proposition 8, it follows that in order to really benefit the overall modularity we must have
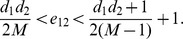
Now we find an upper bound for the RHS inequality. Since 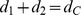, it follows that
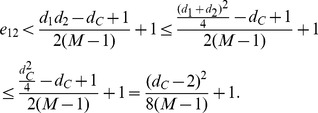
For a lower bound of the LHS inequality, we rewrite 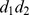 as 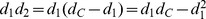 and find the non-zero minimum value on the range 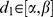. In this interval, 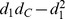 is minimized either at 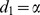 or 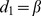. Therefore,
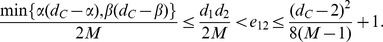
That concludes the proof.□

Finally, our QCA framework is presented in Table 5 Algorithm 5.

**Table 5:** Algorithm 5. **Q**uick **C**ommunity **A**daptation (QCA) Framework.

**Input:** *G*≡*G*_0_ = (*V*_0_, *E*_0_), 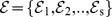 a collection of simple events
**Output:** Community structure 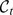 of *G^t^* at time *t*.
1: Use [8] to find an initial community clustering 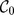 of *G*_0_;
2: **for** (*t* ← 1 to s) **do**
3: 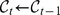 ;
4: **if** ( 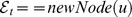 ) **then**
5: Handle 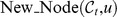 ;
6: **else if** ( 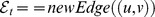 ) **then**
7: Handle 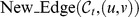 ;
8: **else if** ( 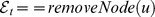 ) **then**
9: Handle 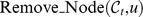 ;
10: **else**
11: Handle 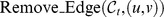 ;
12: **end if**
13: **end for**

## Results

In this section, we first validate our approaches on different synthesized networks with known groundtruths, and then present our findings on real world traces including the Enron email [13], arXiv eprint citation [14], and Facebook social networks [15]. To certify the performance of our algorithms, we compare QCA to three notable adaptive methods including (1) MIEN algorithm proposed by Thang et al. [16], (2) FacetNet framework proposed by Lin et al. [17], and (3) OSLOM method suggested by Lancichinetti et al. [18].

### Results on synthesized networks

Of course, the best way to evaluate our approaches is to validate them on real networks with known community structures. Unfortunately, we often do not know that structures beforehand, or such structures cannot be easily mined from the network topology. Although synthesized data might not reflect all the statistical properties of real networks, they do provide us embedded groundtruths via planted communities, and the ability to vary other parameters such as sizes, densities and overlapping levels, etc. Testing community detection methods on generated data has become an common practice widely accepted in the field [4]. Hence, a comparison between QCA and other dynamic algorithms on synthesized data not only certifies its performance but also provides us the confidence to its behaviors on real world traces.

#### Setup

We use the well-known LFR benchmark [4] to generate 40 networks with 10 snapshots. Parameters are: the number of nodes 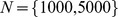, the mixing parameter 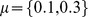 controlling the overall sharpness of the community structure. The experiments are averaged over 1000 runs for consistency. In order to quantify the similarity between the identified communities and the ground truth, we adopt a well known measure in Information Theory called *Normalized Mutual Information (NMI)*. NMI has been proven to be reliable and is currently used in testing community detection algorithms [4]. 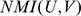 equals 1 if structures *U* and *V* are identical and equals 0 if they are totally separated, and the higher NMI the better. Due to space limit, the readers are encouraged to read [4] for NMI formulas.

#### Results

**Figure 3:**
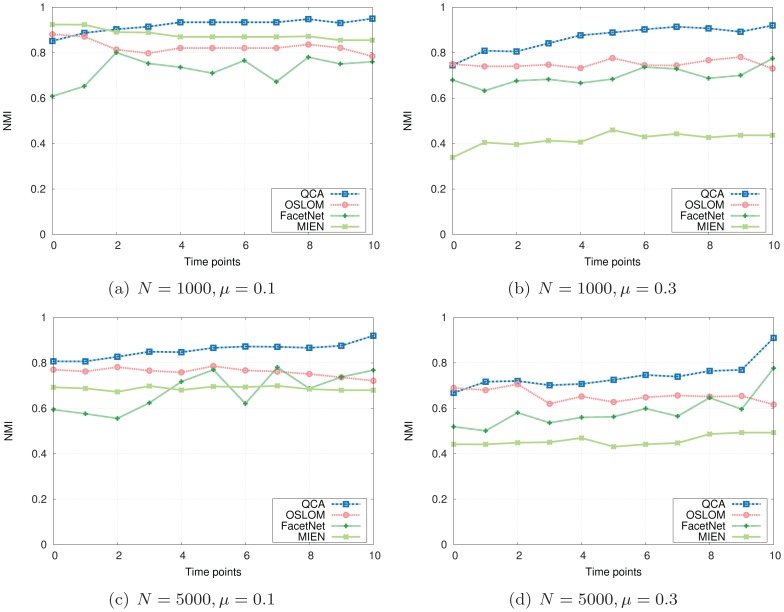
NMI scores on synthesized networks with known communities.

**Figure 4:**
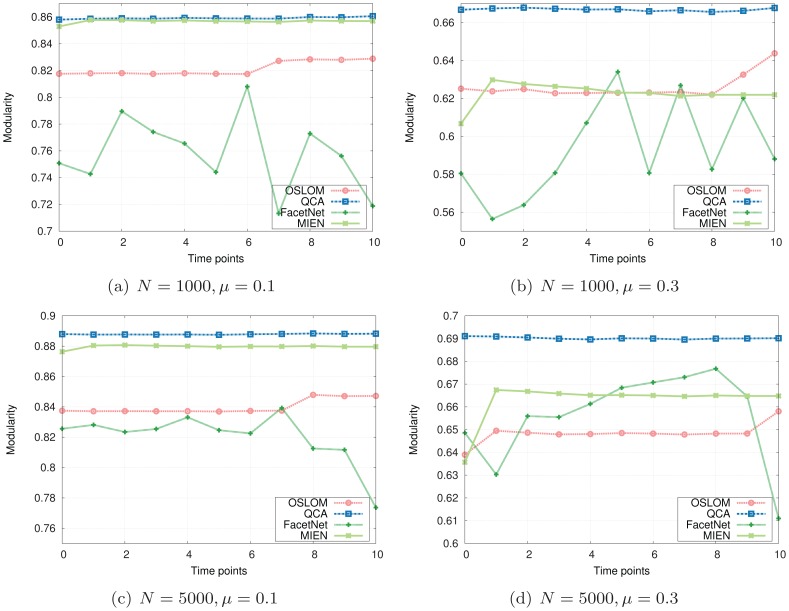
Modularity values on synthesized networks with known communities.

The NMI and Modularity values are reported in Figures 3 and 4. As depicted in their subfigures, the NMI values and modularities indicated by our QCA method, in general, are very high and competitive with those of OSLOM while are much better than those produced by MIEN and FacetNet methods. On these generated networks, we observe that MIEN and FacetNet perform well when the mixing parameter *μ* is small, i.e., when the network community structures are clear, however, their performances degrade dramatically when these structures become less clear as *μ* gets larger. Particularly, MIEN' and FacetNet' NMI scores and modularities in all test cases are fairly low and usually from 10% to 50% and 5% to 15% worst than those produced by QCA. This implies the network communities revealed by these methods are not as high similarity to the ground-truth as QCA algorithm. On the generated networks, OSLOM algorithm performs very well as suggested through its high NMI scores and modularity values. In particular, OSLOM tends to perform better than QCA in the first couple of network snapshots, however, its performance is taken over by QCA when the networks evolve over time, especially at the end of the evolution where OSLM reveals big gaps in similarity to the planted network communities (Note that the higher NMI score at the end of the evolution, the better the final detected community structure). This concludes that the network communities discovered by QCA are of the best similarity to ones planted in the ground-truth in comparison with other methods.

### Results on real-world traces

We next present the results of QCA algorithms on real world dynamic social networks including ENRON email [13], arXiv e-print citation [14], and Facebook networks [15]. Due to the lack of appropriate communities corresponding to these traces, we report the performance of the aforementioned algorithms in reference to the static method proposed by Blondel et al. [8]. In particular, we will show the following quantities (1) modularity values, (2) the quality of the identified network communities through NMI scores, and (3) the processing time of our QCA in comparison with other methods. The above networks possess to contain strong community structures due to their high modularities, which was the main reason for them to be chosen.

For each network, time information is first extracted and a portion of the network data (usually the first snapshot) is then collected to form the basic network community structure. Our QCA method (aslo MIEN and OSLOM) take into account that basic community structure and run on the network changes whereas the static method has to be performed on the whole network snapshot for each time point. In this experiment, FacetNet method does not appear to complete the tasks in a timely manner, and is thus excluded from the plots.

#### ENRON email network

The Enron email network contains email messages data from about 150 users, mostly senior management of Enron Inc., from January 1999 to July 2002 [13]. Each email address is represented by an unique ID in the dataset and each link corresponds to a message between the sender and the receiver. After a data refinement process, we choose 50% of total links to form a basic community structure of the network with 7 major communities, and simulate the network evolution via a series of 21 growing snapshots.

#### Results

**Figure 5:**
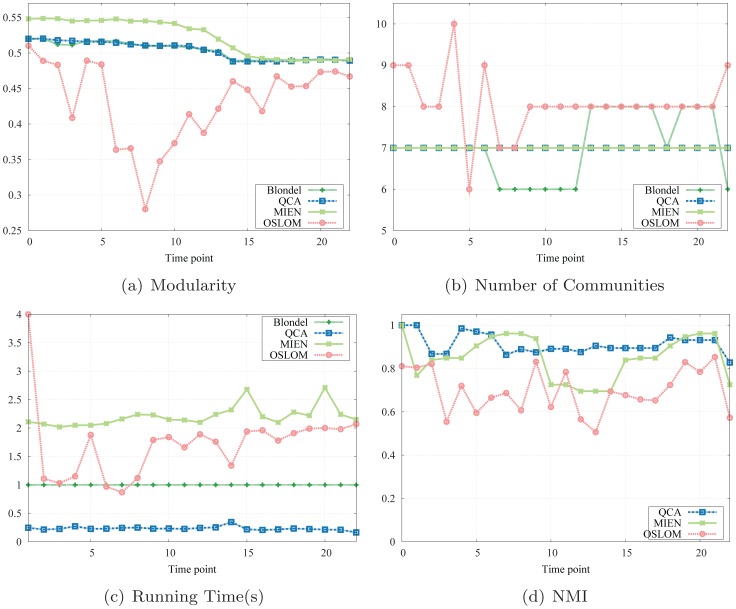
Simulation results on Enron email network.

We first evaluate the modularity values computed by QCA, MIEN, OSLOM, and Blondel methods. As shown in Figure 5a, our QCA algorithm archives competitively higher modularities than the static method but a little bit less than MIEN, and is far better than those obtained by OSLOM. Moreover, QCA also successes in maintaining the same numbers of communities of the other two methods MIEN and Blondel while OSLOM's are vague (Figure 5b). In particular, the modularity values produced by QCA very well approximate those found by static method with lesser variation. There are reasons for that. Recall that our QCA algorithm takes into account the basic community structures detected by the static method (at the first snapshot) and processes on network changes only. Knowing the basic network community structure is a great advantage of our QCA algorithm: it can avoid the hassle of searching and computing from scratch to update the network with changes. In fact, QCA uses the basic structure for finding and quickly updating the local optimal communities to adapt with changes introduced during the network evolution.

The running time of QCA and the static method in this small network are relatively close: the static method requires one second to complete each of its tasks while our QCA does not even ask for one (Figure 5c). In this dataset, MIEN and OSLOM requires a little more time (1.5 and 2.4 seconds in average for MIEN and OSLOM) to complete their tasks. Time and computational cost are significantly reduced in QCA since our algorithms only take into account the network changes while the static method has to work on the whole network every time.

As reported in Figure 5d, both the NMI scores of ours and MIEN method are very high and relatively close to 1 while those obtained by OSLOM fall short and are far from stable. These results indicate that in this Enron email network, both QCA and MIEN algorithms are able to identify high quality community structure with high modularity and similarity; however, only our method significantly reduces the processing time and computational requirement.

#### arXiv e-print citation network

The arXiv e-print citation network [14] has become an essential mean of assessing research results in various areas including physics and computer sciences. This network contained more than 225K articles from January 1996 to May 2003. In our experiments, citation links of the first two years 1996 and 1997 were used to form the basic community structure of our QCA method. In order to simulate the network evolution, a total of 30 time dependent snapshots are created on a two-month regular basis from January 1998 to January 2003.

#### Results.

**Figure 6:**
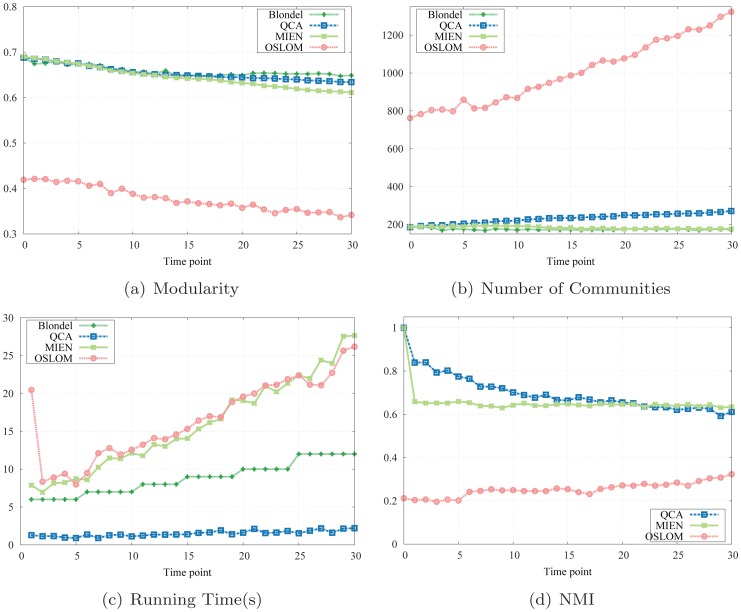
Simulation results on arXiv e-print citation network.

We compare modularity results obtained by QCA algorithm at each network snapshot to Blondel as well as to MIEN and OSLOM methods. It reveals from Figure 6a that the modularities returned by QCA are very close to those obtained by the static method with much more stabler and are far higher than those obtained by OSLOM and MIEN. In particular, the modularity values produced by QCA algorithm cover from 94% up to 100% that of Blondel method and from 6% to 10% higher than MIEN and at least 1.5× better than OSLOM. In this citation networks, the numbers of communities detected by OSLOM take off with more than 1200 whereas those found by QCA, MIEN and Blondel methods are relatively small (Figure 6b). Our QCA method discovers more communities than both Blondel and MIEN as the network evolves and this can be explained based on the resolution limit of modularity [19]: the static method might disregard some small communities and tend to combine them in order to maximize the overall network modularity.

A second observation on the running time shows that QCA outperforms the static method as well as its competitor MIEN: QCA takes at most 2 seconds to complete updating the network structure while Blondel method requires more than triple that amount of time, MIEN and OSLOM asks for more than 5 times (Figure 6c). In addition, higher NMI scores of QCA than MIEN's and especially OSLOM's scores (Figure 6d) implies network communities identified by our approach are not only of high similarity to the ground truth but also more precise than that detected by MIEN, while the computational cost and the running time are significantly reduced.

#### Facebook social network

This dataset contains friendship information among New Orleans regional network on Facebook [15], spanning from September 2006 to January 2009 with more than 60K nodes (users) connected by more than 1.5 million friendship links. In our experiments, nodes and links from September 2006 to December 2006 are used to form the basic community structure of the network, and each network snapshot is recored after every month during January 2007 to January 2009 for a total of 25 network snapshots.

#### Results

**Figure 7:**
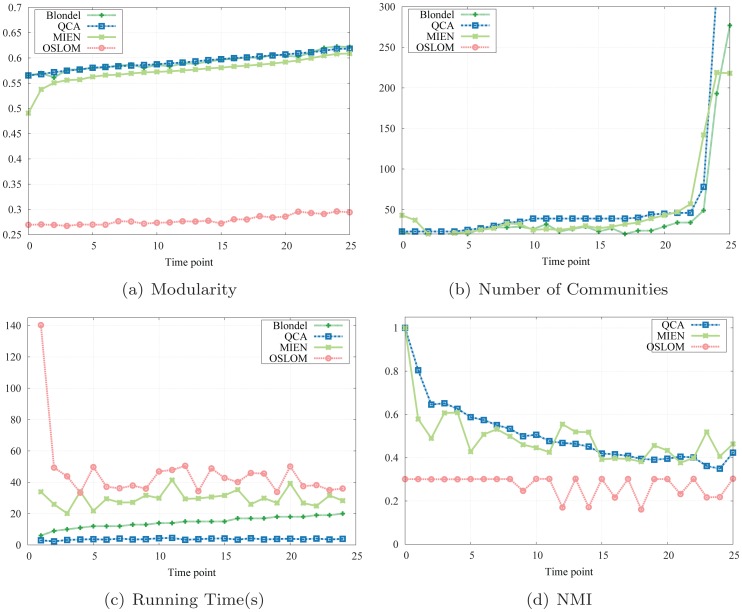
Simulation results on Facebook social network.

The evaluation depicted in Figure 7a reveals that QCA algorithm achieves competitive modularities in comparison with the static method, and again far better than those obtained by MIEN and OSLOM method, especially in comparison with OSLOM whose perform was nice on synthesized networks. In the general trend, the line representing QCA results closely approximates that of the static method with much more stability. Moreover, the two final modularity values at the end of the experiment are relatively the same, which means that our adaptive method performs competitively with the static method running on the whole network.

Figure 7c describes the running time of the three methods on the Facebook data set. As one can see from this figure, QCA takes at least 3 seconds and at most 4.5 seconds to successfully compute and update every network snapshot whereas the static method, again, requires more than triple processing time. MIEN and OSLOM methods really suffer on this large scale network when requiring more than 10× and 11× that amounts of QCA running times. In conclusion, high NMI and modularity scores together with decent executing times on all test cases confirm the effectiveness of our adaptive method, especially when applied to real world social networks where a centralized algorithm, or other dynamic algorithms, may not be able to detect a good network community structure in a timely manner.

However, there is a limitation of QCA algorithm we observe on this large network and want to point out here: As the the duration of network evolution lasts longer over time (i.e., the number of network snapshots increases), our method tends to divide the network into smaller communities to maximize the local modularity, thus results in an increasing number of communities and a decreasing of NMI scores. Figure 7b and 7d describes this observation. For instance, at snapshot 12 (a year after December 2006), the NMI score is approximately 1/2 and continues decaying after this time point. It implies a refreshment of network community structure is required at this time, after a long enough duration. This is reasonable since activities on an online social network tend to come and go rapidly and local adaptive procedures are not enough to reflect the whole network topology over a long period of time.

### A social-aware message forwarding strategy in MANETs

In this section, we present a practical application where the detection of network community structures plays an important role in routing strategies in MANETs. A MANET is a dynamic wireless network with or without the underlying infrastructure, in which each node can move freely in any direction and organize itself in an arbitrary manner. Due to nodes mobility and unstable links nature of a MANET, designing an efficient routing scheme has become one of the most important and challenging problems on MANETs.

Recent researches have shown that MANETs exhibit the properties of social networks [20]–[22] and social-aware algorithms for network routing are of great potential. This is due to the fact that people have a natural tendency to form groups or communities in communication networks, where individuals inside each community communicate with each other more frequent than with people outside. This social property is nicely reflected to the underlying MANETs by the existence of groups of nodes where each group is densely connected inside than outside. This resembles the concept of *community structure* in Mobile Ad hoc Networks.

Multiple routing strategies [21], [23] based on the discovery of network community structures have provided significant enhancement over traditional methods. However, the community detection methods utilized in those strategies are not applicable for dynamic MANETs since they have to recompute the network structure whenever changes to the network topology are introduced, which results in significant computational costs and processing time. Therefore, employing an adaptive community structure detection algorithm as a core will provide a speedup as well as robust to routing strategies in MANETs.

We evaluate five routing strategies (1) WAIT: the source node waits until it meets the destination node (2) MCP: A node keeps forwarding the messages until they reach the maximum number of hops (3) LABEL: A node forwards or sends the messages to all members in the destination community [20] (4) QCA: A Label version utilizing QCA as the dynamic community detection method and lastly, (5) MIEN: A social-aware routing strategy on MANETs [16].

Even though WAIT and MCP algorithms are very simple and straightforward to understand, they provide us helpful information about the lower and upper bounds on the message delivery ratio, time redundancy as well as message redundancy. The LABEL forwarding strategy works as follow: it first finds the community structure of the underlying MANET, assigns each community with the same label and then exclusively forwards messages to destinations, or to next-hop nodes having the same labels as the destinations. MIEN forwarding method utilizes MIEN algorithm as a subroutine. QCA routing strategy, instead of using a static community detection method, employs QCA algorithm for adaptively updating the network community structure and then uses the newly updated structure to inform the routing strategy for forwarding messages.

We choose Reality Mining data set [24] provided by the MIT Media Lab to test our proposed algorithm. The Reality Mining data set contains communication, proximity, location, and activity information from 100 students at MIT over the course of the 2004–2005 academic year. In particular, the data set includes call logs, Bluetooth devices in proximity, cell tower IDs, application usage, and phone status (such as charging and idle) of the participated students of over 350,000 hours (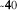 years). In this paper, we take into account the Bluetooth information to form the underlying MANET and evaluate the performance of the above five routing strategies.

**Figure 8:**
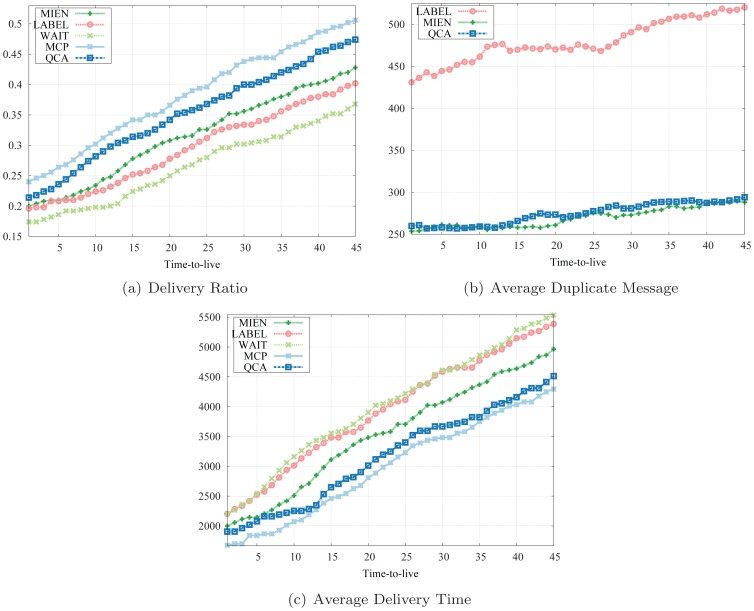
Experimental results on the Reality Mining data set.

For each routing method, we evaluate the followings (1) Delivery ratio: The portion of successfully delivered over the total number of messages (2) Average delivery time: Average time for a message to be delivered. (3) Average number of duplicated messages for each sent message. In particular, a total of 1000 messages are created and uniformly distributed during the experiment duration and each message can not exist longer than a threshold *time-to-live*. The experimental results are shown in Figure 8a, 8b and 8c.

#### Results

Figure 8a describes the delivery ratio as a function of *time-to-live*. As revealed by this figure, QCA achieves much better delivery ratio than MIEN as well as LABEL and far better than WAIT. This means that QCA routing strategy successfully delivers many more messages from the source nodes to the destinations than the others. Moreover, as *time-to-live* increases, the delivery ratio of QCA tends to approximate the ratio of MCP, the strategy with highest delivery ratio.

Comparison on delivery time shows that QCA requires less time and gets messages delivered successfully faster than LABEL, as depicted in Figure 8c. It even requires less delivery time in comparison with the social-aware method MIEN. This can be explained as the static community structures in LABEL can possibly get message forwarded to a wrong community when the destinations eventually change their communities during the experiment. Both QCA and MIEN, on the other hand, captures and updates the community structures on-the-fly as changes occur, thus achieves better results.

The numbers of duplicate messages presented in Figure 8b indicate that both QCA and MIEN achieves the best results. The numbers of duplicated messages of MCP method are substantially higher than those of the others and are not plotted. In fact, the results of QCA and MIEN are relatively close and tend to approximate each other as *time-to-live* increases.

In conclusion, QCA is the best social-aware routing algorithm among five routing strategies since its delivery ratio, delivery time, and redundancy outperform those of the other methods and are only below MCP while the number of duplicate messages is much lower. QCA also shows a significant improvement over the naive LABEL method which uses a static community detection method and thus, confirms the applicability of our adaptive algorithm to routing strategies in MANETs.

### Worm containment in social networks

In this section, we present a practical application of QCA method in Worm Containment in OSNs. Since their introduction, popular social network sites such as Facebook, Twitter, Bebo, and MySpace have attracted millions of users worldwide, many of whom have integrated those sites into their everyday lives. On the bright side, OSNs are ideal places for people to keep in touch with friends and colleagues, to share their common interests, or just simply to socialize online. However, on the other side, social networks are also fertile grounds for the rapid propagation of malicious softwares (such as viruses or worms) and false information.

Facebook, one of the most famous social sites, experienced a wide propagation of a trojan worm named “Koobface” in late 2008. Koobface made its way not only through Facebook but also Bebo, MySpace and Friendster social networks [25] [26]. Once a user's machine is infected, this worm scans through the current user's profile and sends out fake messages or wall posts to everyone in the user's friend list with titles or comments to appeal to people's curiosity. If one of the user's friends, attracted by the comments without a shadow of doubt, clicks on the link and installs the fake “flash player”, his computer will be infected and Koobface's life will then cycle on this newly infected machine.

Worm containment problem becomes more and more pressing in OSNs as this kind of networks evolves and changes rapidly over time. The dynamics of social networks thus gives worms more chances to spread out faster and wider as they can flexibly switch between existing and new users in order to propagate. Therefore, containing worm propagation on social networks is extremely challenging in the sense that a good solution at the previous time step might not be sufficient or effective at the next time step. Although one can recompute a new solution at each time the network changes, doing so would result in heavy computational costs and be time consuming as well as allowing worms spreading out wider during the recomputing process. A better solution should quickly and adaptively update the current containing strategy based on changes in network topology, and thus can avoid the hassle of recomputation.

There are many proposed methods for worm containment on computer networks by either using a multi-resolution approach [27], or using a simplification of the Threshold Random Walk scan detector [28], or using fast and efficient worm signature generation [29]. There are also several methods proposed for cellular and mobile networks [30] [31]. However, these approaches fail to take into account the community structure as well as the dynamics of social networks, and thus might not be appropriate for our problem. A recent work [3] proposed a social-based patching scheme for worm containment on cellular networks. However, this method encounters the following limitations on a real social network (1) its clustered partitions do not necessarily reflect the natural network communities, (2) it requires the number of clusters *k* (which is generally unknown for social networks) must be specified beforehand, and (3) it exposes weaknesses when dealing with the network's dynamics.

To overcome these limitations, our approach first utilizes QCA to identify the network community structure, and adaptively keeps this structure updated as the network evolves. Once network communities are detected, our patch distribution procedure will select the most influential users from different communities in order to send patches. These users, as soon as they receive patches, will apply them to first disinfect the worm and then redistribute them to all friends in their communities. These actions will contain worm propagation to only some communities and prevent it from spreading out to a larger population. To this end, a quick and precise community detection method will definitely help the network administrator to select a more sufficient set of critical users to send patches, thus lowering down the number of sent patches as well as overhead information over the social network.

We next describe our patch distribution. This procedure takes into account the identified network communities and selects a set of influential users from each community in order to distribute patches. *Influential users* of a community are ones having the most relationships or connections to other communities. In an adversary point of view, these influential users are potentially vulnerable since they not only interact actively within their communities but also with people outside, and thus, they can easily fool (or be fooled by) people both inside and outside of their communities. On the other point of view, these users are also the best candidates for the network defender to distribute patches since they can easily announce and forward patches to other members and non-members. In Table 6 Algorithm 6, we present a quick algorithm for selecting the set of most influential users in each community. This algorithm starts by picking the user whose number of social connections to outside communities is the highest, and temporarily disregards this user from the considering community. This process repeats until no connections crossing among communities exists. This set of influential users is the candidate for the network defender for distributing patches.

**Table 6:** Algorithm 6. Patch Distribution Algorithm.

**Input:** *G* = (*V*, *E*) and its community structure 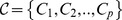
**Output:** The set of influential users 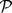 .
1: 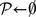 ;
2: **for** 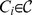 **do**
3: **while** ( 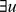 unvisited in *C_i_* satisfying 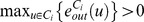 ) **do**
4: Let 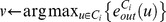 ;
5: 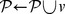 ;
6: Mark *v* as visited in *C_i_*;
7: **end while**
8: **end for**
9: Send patches to users in 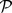 ;

### Experimental results

We present the results of our QCA method on the Facebook network dataset [15] and compare the results with the social based method (Zhu's method [3]) via a weighted version of our algorithms. One notable feature of this dataset is time information (stamped at every moment the information was recorded) representing the dynamics of the network, which nicely suits our method.

#### Set up

The worm propagation model in our experiments mimics the behavior of the famous “Koobface” worm, i.e., worms are able to explore their victim's friend list and then send out fake messages containing malicious links for propagating. The probabilities of activating the worm is proportional to communication frequency between the victim and his friends. The time taken for worms to spread out from one user to another is inversely proportional to the communication frequency between this user and his particular friend. Finally, when a worm has successfully infected a user's computer, it will start propagating as soon as this computer connects to a specific social network (Facebook in this case). When the fraction of infected users reaches a threshold *α*, the detection system raises an alarm and patches will automatically be sent to most influential users selected by Table 6 Algorithm 6. Once a user receives the patch, he will first apply it to disinfect the worm and then will have an option to forward it to all friends in his community. Each experiment is seeded with 0.02% of users to be initially infected by worms.

We compare infection rates of the social-based method of Zhu's and ours. The infection rate is computed as the fraction of the remaining infected users over all infected ones. The number of clusters *k* in Zhu's method is set to be 150 in static and 200 in dynamic networks, and for each value of *k*, the alarming threshold *α* is set to be 2%, 10%, and 20%, respectively. Each experiment is repeated 1000 times for consistency.

#### Result

**Figure 9:**
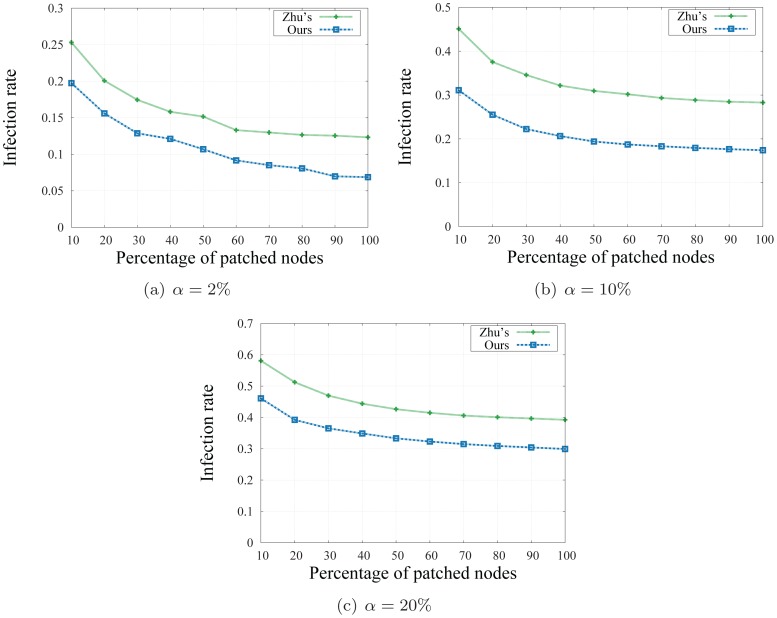
Infection rates on static network with *k* = 150 clusters.

Figure 9, 10 show the results of our experiments for three different values of *k* and *α*. We first observe that the longer we wait (the higher the alarm threshold is), the higher number of users we need to send patches to in order to achieve the desired infection rate. For example, with *k* = 150 clusters and an expected infection rate of 0.3, we need to send patches to less than 10% number of users when *α* = 2%, to more than 15% number of users when *α* = 10% and to nearly 90% of total influential users when *α* = 20%.

**Figure 10:**
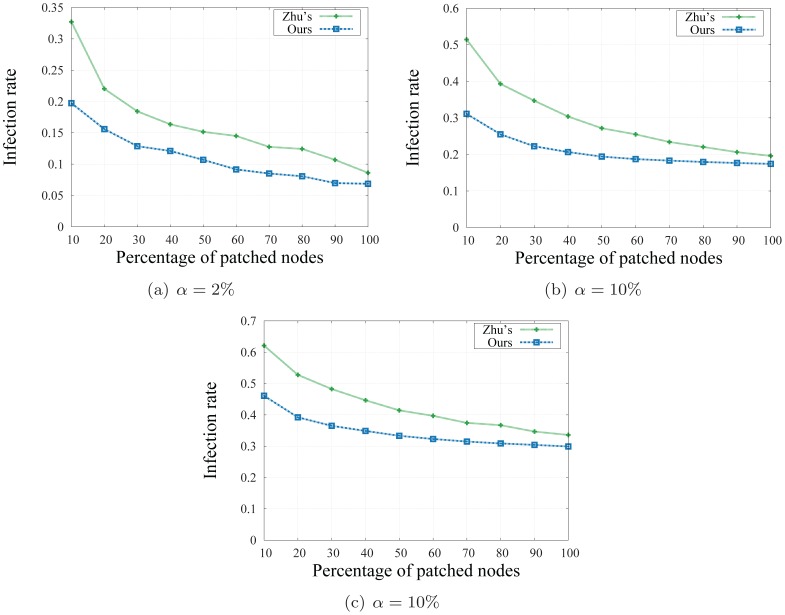
Infection rates on dynamic network with *k* = 200 clusters.

A second observation reveals that our approach achieves better infection rates than the social-based method of Zhu's in a static version of the social network as depicted in Figure 9. In particular, the infection rates obtained in our method are from 5% to 10% better than those of Zhu's. When the network evolves as new users join in and new social relationships are introduced, we resize the number of cluster *k* and recompute the infection rates of the social based method with the number of cluster *k* = 200, and the alarm threshold *α* = 2% and 10% respectively. As depicted in Figures 10, our method, with the power of quickly and adaptively updating the network community structure, achieves better infection rates than Zhu's method while the computational costs and running time is significantly reduced. As discussed, detecting and updating the network community is the crucial part of a social based patching scheme: a good and up-to-date network community structure will provide the network defender a tighter set of vulnerable users, and thus, will help to achieve lower infection rates. Our adaptive algorithm, instead of recomputing the network structure every time changes are introduced, quickly and adaptively updates the network communities on-the-fly. Thanks to this frequently updated community structure, our patch distribution procedure is able to select a better set of influential users, and thus helps in reducing the number of infected users once patches are sent.

Finally, a comparison on running time on the two approaches shows that time taken for Zhu's method is much more than our community updating procedure, and hence, may prevent this method to complete in a timely manner. In particular, our approach takes only 3 seconds for obtaining the basic community structure and at most 30 seconds to complete all the tasks whereas [3] requires more than 5 minutes to divide the communication network into modules and selecting the vertex separators. In that delay, worm propagation may spread out to a larger population, and thus, the solution may not be effective. These experimental results confirm the efficiency of our approach on social networks.

## Related work

Community detection on static networks has attracted a lot of attentions and many efficient methods have been proposed for this type of networks [32]. Detecting community structure on dynamic networks, however, has so far been an untrodden area. In [33], the authors defined time graphs that captured the link creation as a point phenomena in time of a directed evolving graph, and studied the evolution of the blogosphere in terms of changes such as in-degree, out-degree, etc. Another work [34] studied the growth of the a wide range of real-world evolving graphs and provided a new kind of graph generator that produced networks with the discovered patterns. In [35], the authors suggested a method for observing the evolution of web communities by first revealing network communities at each time point, and then quantifying changes that occurred to network communities based on community changes such as emerging, growing and shrinking.

One of the most seminal work [2] proposed an innovative method for detecting communities on dynamic networks the based on *k*-clique percolation technique. This approach can detect overlapping nodes in different network communities; however, its internal *k*-clique percolation technique may require high computing resources and thus, may be time consuming especially on large OSNs. A work in [13] presented GraphScope, a parameter-free method for detecting clusters on time-evolving graphs based on mutual information and entropy functions. However, it requires a recomputation of the number of sources and destinations each time the graph segments change without utilizing its previously computed information. Thus, it might not lend itself effectively to the field of adaptive algorithms. [36] attempted to track the evolving of communities over time, using a few static network snapshots.

A recent work of [37] proposed a detection method based on contradicting the network topology and the topology-based *propinquity* - the probability of a pair of nodes involved in a community. Another attempt which is closely related to our work includes [38] in which the authors proposed *FacetNet*, a framework to track community evolutions in a unified process. In this framework, the community structure at a given time step is found both by the observed the network data and the prior distribution given by historic community structures. A limit of this framework is that at each time step, the underlying algorithm should be executed for multiple values of *m*-the number of communities, which might prevent this framework from being effective on real world social networks.

The authors [39] present a framework for detecting dynamic communities with a constant factor approximation. This property is nice, however, this method also requires some predefined costs to penalize people moving in or out of a community, which might be generally unknown in dynamic social networks. A recent work [16] proposes a social-aware routing strategy, named MIEN, which also makes uses of a modularity-based procedure for quickly updating the network structure. In particular, MIEN tries to compose and decompose network modules in order to keep up with the changes and uses fast modularity algorithm [5] to update the network modules. However, this method may be time consuming due to the high complexity of [5].

## Conclusions

We presented QCA, an adaptive method for detecting and tracing community structures in dynamic social networks. We show that our adaptive method is not only effective in identifying high quality network community structures, but also has the great advantage of fast running time, which is suitable for large OSNs. We prove some theoretical results which are the basic observations of our approach. Finally, via practical applications in forwarding and routing stategies in MANETs and worm containment on social networks, we show that our QCA method promises a wide range of real applications not only on mobile computing but also on OSNs as it can be deployed into many community detection modules.
